# An adult case of large defect of small mesenteric hiatal hernia causing small bowel obstruction

**DOI:** 10.1186/s40792-024-02040-3

**Published:** 2024-10-28

**Authors:** Yuki Horiuchi, Erica Nishimura, Eriko Sashi, Kazuhiro Matsuo, Nozomi Watanobe, Risa Ohtani, Koshiro Matsunami, Takako Muroi, Asuka Hara, Keita Hayashi, Yuki Tajima, Yasushi Kaneko, Rurika Hamanaka, Hiroto Fujisaki, Kumiko Hongo, Kikuo Yo, Kimiyasu Yoneyama, Kiminori Takano, Motohito Nakagawa

**Affiliations:** Hiratsuka City Hospital, 1-19-1 Minamihara, Hiratsuka-Shi, Kanagawa 254-0065 Japan

**Keywords:** Mesenteric hiatal hernia (congenital transmesenteric hernia), Internal hernia, Strangulated intestinal obstruction, Small bowel obstruction

## Abstract

**Background:**

Small mesenteric hiatal hernias (SMHHs) are defined as a small group of internal hernias (IHs) that frequently diagnosed in children. However, SMHHs are relatively rare in adults. Bowel loop herniation via an abnormal mesenteric defect can lead to strangulated intestinal obstruction. Congenital SMHHs are commonly observed in pediatric patients, with some cases involving neonatal death.

**Case presentation:**

A 24-year-old healthy male patient visited our hospital with a 2-day history of a sudden onset lower abdominal pain. He was initially diagnosed with enteritis. However, his symptoms worsened, and he was brought to our hospital. Contrast-enhanced computed tomography (CT) scan showed formation of a closed loop in the small intestine within the pelvis and signs of ischemia. As the patient was diagnosed with small bowel obstruction (SBO) caused by IH, emergency laparoscopic surgery was performed to loosen the obstruction. The patient was found to have ascites and small-bowel necrosis. A part of the small intestine that measured 30 cm was strangulated via a large-diameter defect (17 × 11 cm) in the ileal mesentery. Via a small abdominal incision, the necrotic bowel was resected, and the mesenteric defect was repaired.

**Conclusion:**

SMHHs are rare in adults, and they should be considered as potential causes of strangulated intestinal obstruction in adults without a history of laparotomy or trauma.

## Background

Internal hernias (IHs) are defined as the protrusion of the intestine via an aperture in the peritoneal ligament, mesentery, or omentum. IHs are classified into two groups: herniation via an intraperitoneal fossa or cystic lump (40%) and herniation via an abnormal hiatus located on the mesentery, omentum, or broad ligament of the uterus (60%). Takahashi et al. showed that IH was relatively rare, accounting for 0.01%–5% of all small bowel obstruction (SBO) cases [1, 2].

Mesenteric hiatal hernias are diseases classified based on abnormal mesenteric hiatus causing SBO. In particular, they are divided into small mesenteric hiatal hernias (SMHHs) accounting for 70%, and colon mesenteric hiatal hernias accounting for 30%, respectively [1]. In the study of Sunami et al., according to hernia location, the small intestinal mesenteric hiatus (64%) is the most common site, followed by the sigmoid colon mesentery (22.4%), transverse colon mesentery (12.2%), ascending colon mesentery (0.7%), and descending colon mesentery (0.7%) [3]. In general, SMHHs are usually observed in the neonatal or pediatric age group (80%), and adult SMHH cases are relatively rare [4]. Bowel loop herniation via an abnormal mesenteric defect can lead to strangulated intestinal obstruction.

SMHHs are frequently diagnosed during surgery. Congenital pediatric SMHHs are commonly reported, with neonatal death also noted in some cases. According to the study of Newsom et al., congenital IHs causing SBO and intestinal gangrene are associated with a 50% mortality rate because of delayed presentation and diagnosis [5]. Meanwhile, in 0.2%–0.5% of cases, mesenteric hiatus was observed during autopsy. Thus, it does not necessarily cause SBO [4].

Herein, we report a 24-year-old male patient who presented with severe sudden-onset abdominal pain and who was later diagnosed with intestinal gangrene due to a large-diameter mesenteric hiatal hernia.

## Case presentation

A 24-year-old male patient with a complaint of sudden-onset lower abdominal pain and nausea visited a local clinic. The patient did not present with fever, constipation, and diarrhea. He was initially diagnosed with enteritis at the clinic. However, the symptoms worsened within the next 2 days. Thus, the patient was brought to our emergency department. The patient had no similar episode of abdominal pain and had no history of abdominal surgeries or trauma or medication use.

The patient experienced severe abdominal pain and tenderness, and guarding was observed. He was hemodynamically stable, with a temperature of 37.2 °C, blood pressure of 134/69 mmHg, heart rate of 108 bpm, and respiratory rate of 24 beats per minute. Further, he presented with adequate oxygen saturation on room air. His blood tests results indicated leukocytosis and dehydration (Table 1).

**Table 1:** Blood examination results

Parameters	Patient values	Unit
WBC count	11,100	/μL
Hb level	17.1	g/dL
Platelet count	231	× 10^3^/μL
Sodium level	134	mEq/L
Potassium level	4.0	mEq/L
Chloride level	93	mEq/L
Total protein level	7.6	g/dL
Albumin level	4.6	g/dL
BUN level	23	mg/dL
Creatinine level	0.85	mg/dL
Total bilirubin level	1.6	mg/dL
Direct bilirubin level	0.5	mg/dL
AST level	26	U/L
ALT level	16	U/L
LD level	186	U/L
γ-GT level	21	U/L
CK level	172	U/L
CRP level	0.72	mg/dL
pH	7.620	
pO2	28.9	mmHg
pCO2	24.9	mmHg
HCO3	25.6	mmol/L
BE	6.0	mmol/L
Anion gap	16.7	mmol/L

**Fig. 1:**
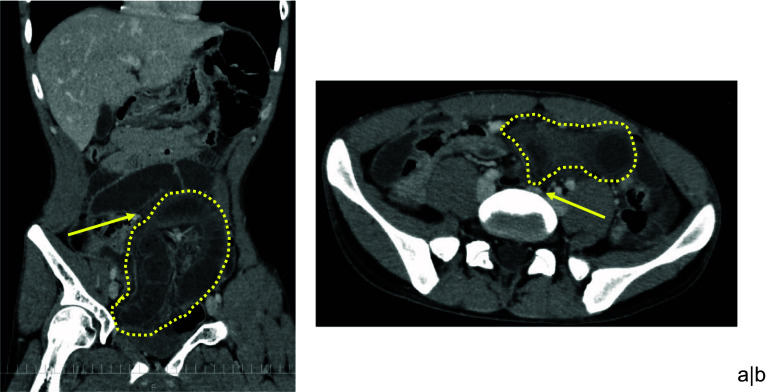
CT scan findings. **a** A closed loop (dotted line) formed in the left lower abdomen. The obstruction point was at the midline of the abdomen (arrow). **b** Contrast-enhanced imaging showed intestinal edema with a partially non-enhanced area. A closed loop (dotted line) and the site of obstruction (arrow) were identified

Radiography and contrast-enhanced computed tomography (CT) scan were performed. The radiographic image revealed a small intestinal gas. However, there was no significant appearance of free air and air-fluid levels. CT scan showed the formation of a closed loop in the small bowel. The intestinal walls were thick, edematous, and not partially enhanced. A moderate volume of bloody ascites was also identified in the pelvis (Fig. 1). According to the clinical examination and CT scan findings, the patient was diagnosed with strangulated intestinal obstruction due to IH. Thus, emergency laparoscopic surgery was performed.

**Fig. 2:**
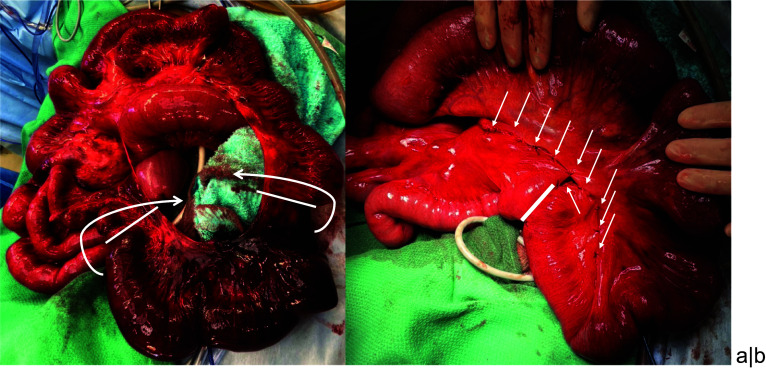
Intraoperative findings. **a** The necrotic bowel loop herniated via the large-diameter mesenteric defect, twisting along the arrows. The solid lines show the position of the bowel resection. The upper side of the image presents the cranial side. **b** The mesenteric defect was repaired after resecting the necrotic bowel. The solid line is the point of the functional end-to-end anastmosis. The white arrows refer the suturing point to close the rest mesenteric defect. The upper side of the image shows the cranial side

Emergency exploratory laparotomy revealed gangrenous ileal segment along with bloody ascites in the pelvis. As the patient’s necrotic bowels needed to be resected, a small incision was made on the abdomen, and the whole intestine was assessed. The necrotic intestine herniated via a large-diameter defect, measuring 17 × 11 cm, in the ileal mesentery (Fig. 2a). The length of the necrotic bowel, which began from 210 cm distal to the Treitz ligament, was 30 cm. The strangulation was repaired, the gangrenous bowel was resected, and functional end-to-end anastomosis was performed. Subsequently, the mesenteric membrane of the defect was sutured by 3–0 soft silk thread. The marginal vessels around the defect were thick; therefore, extra precaution was taken not to damage the vessels and deteriorate the blood flow of the remnant small bowel. The anastomosis and the suture line are described in Fig. 2b.

After the surgery, the patient developed paralytic ileus. However, he had a favorable course without any significant complications and was discharged on the 12th postoperative day.

## Discussion

IHs are classified into congenital and acquired. In most cases, the defect is congenital. This phenomenon is attributed to two reasons: first, IH can develop in a wide range of ages. However, in most cases, it occurs in children. Second, based on intraoperative findings, the defect is usually circular and smooth, and it lacks signs of inflammation. In our case, although the mechanism of formation was unclear, he had no history of abdominal trauma, and the defect’s edges were smooth. In addition, the development of the marginal vessels indicates that the defect enlarged and that it had a chronic course. Hence, the patient’s mesenteric hiatal hernia could be congenital.

SMHH is challenging to diagnose preoperatively. Blachar et al. showed that the characteristic CT scan findings of SMHH include a closed loop in the small intestine, clustered and congested mesenteric vessels, and displacement of the mesenteric vascular trunk [6]. In our case, the patient presented with a closed loop pattern, and strangulated intestinal obstruction caused by the internal hernia was suspected. However, the actual cause was not identified preoperatively. This emphasizes diagnostic difficulties in this condition without specific considerations.

Although there are a few documented and published case reports, systematic reviews on the presentation, ideal management, and outcomes of IH are not available. A search for studies on small mesenteric hiatus hernias from 1983 to 2023, which excluded conference proceedings, revealed 29 adult (aged 20 years and older) cases in Japan [4, 7–32] (Table 2). The mean age of the patients was 48.9 years, and sex differences were not observed. The ileal mesentery is the most common site, and the average defect size was 6.8 (standard deviation: 6.2) cm. The defect in our case was significantly larger than that in other cases. Although there is no specific definition for large-diameter SMHHs, a defect greater than 15 cm was only observed in four cases. According to Ohata et al., the median defect size in pediatric cases is < 4 cm, which is smaller than the average defect size in adults [7]. Therefore, if the mesenteric hiatal defect is large, the patient can avoid strangulated intestinal obstruction in childhood.

**Table 2:** Adult cases of small mesenteric hiatal hernia

Hiatus location	Length of the resected bowel (cm)	Maximum hiatal diameter (cm)	Sex	Age	Year	Author
Mesenteric trunk	260	3	Male	46	1990	Nagashima [8]
Ileocecal mesentery	5	6	Male	54	1991	Narita [9]
Small mesentery	152	N.D	Female	28	1991	Takehara [10]
Ileocecal mesentery	400	1.5	Female	65	1993	Tomikawa [11]
**Central small-bowel mesentery**	**40**	**20**	**Female**	**67**	**1993**	**Kiyasu **[12]
Ileocecal mesentery	120	2	Male	74	1994	Sato [13]
Small mesentery	No resection	5	Female	20	1997	Nozaki [14]
Ileal mesentery	150	N.D	Female	22	1998	Ogizawa [15]
Ileocecal mesentery	70	N.D	Female	61	1999	Kayama [16]
Ileal mesentery	190	3	Female	26	2000	Yanagawa [17]
Ileocecal mesentery	270	2	Male	62	2001	Sato [18]
Ileocecal mesentery	247	5	Male	77	2001	Tahata [19]
Ileocecal mesentery	140	2	Female	75	2002	Kii [20]
Ileocecal mesentery	150	N.D	Male	66	2002	Nobuhara [21]
Ileal mesentery	190	N.D	Male	80	2002	Uenishi [22]
Jejunal mesentery	270	3	Male	33	2002	Hori [4]
Small mesentery	80	6	Male	29	2009	Kunii [23]
Ileal mesentery	No resection	3	Male	21	2012	Kurokouchi [24]
Ileal mesentery	150	4	Female	28	2012	Kurokouchi [24]
**Ileal mesentery**	**170**	**20**	**Female**	**74**	**2012**	**Oshima** [25]
Ileal mesentery	190	11	Female	59	2013	Shirata [26]
Ileal mesentery	150	N.D	Female	40	2013	Hara [27]
Ileal mesentery	No resection	3	Male	39	2014	Koterazawa [28]
Ileal mesentery	100	3	Female	50	2015	Ito [29]
Ileal mesentery	100	10	Male	76	2016	Tokura [30]
Ileal mesentery	100	3	Female	54	2017	Yamazaki [31]
Ileocecal mesentery	47	3	Male	20	2017	Ono [32]
**Ileal mesentery**	**45**	**20**	**Female**	**48**	**2019**	**Takano** [33]
**Ileal mesentery**	**30**	**17**	**Male**	**24**	**2024**	**Our case**

^*^The bold text refers to cases of large-dimeter SMHH (maximum hiatal dimeter ≥ 15 cm)

The surgical techniques for IHs associated with an abnormal hiatus include necrotic bowel resection and defect closure for hernial repair. If bowel necrosis is suspected, bowel resection is required. Hori et al. reported that different surgical techniques can be used for SMHHs [4]. In particular, 102 (76.7%) of 133 patients with SMHHs underwent bowel resection, and 31 (23.2%) patients did not. Further, 63 patients received surgical treatment for an abnormal defect. The defect was resected in 41 (65.1%) cases, and suture closure for defect repair was performed on 22 (34.9%) patients [4]. If the hernia defect is large, suture closure could be beneficial to preserve the margin artery and shorten the length of the bowel that should be resected.

## Conclusion

Considering SMHH as a potential cause of strangulated intestinal obstruction is helpful for diagnosis and treatment in adults without a history of surgery or trauma. In our case, the patient developed SBO due to a large SMHH. Based on this case, patients with a large-diameter defect can avoid SBO in childhood.

## Data Availability

The datasets supporting the conclusions of this article are included within the article.

## Abbreviations

IH: Internal hernia
SMHH: Small mesenteric hiatal hernia
SBO: Small-bowel obstruction
CT: Computed tomography
